# Massage therapy for cancer patients: a reciprocal relationship between body and mind

**DOI:** 10.3747/co.2007.105

**Published:** 2007-04

**Authors:** S.M. Sagar, T. Dryden, R.K. Wong

**Affiliations:** * Juravinski Cancer Program, Hamilton Health Sciences Corporation and McMaster University, Hamilton, Ontario; † Centre for Applied Research, Centennial College, Toronto, Canada.; ‡ McMaster University, Departments of Oncology and Medicine, Hamilton, Ontario

**Keywords:** Massage, cancer, clinical trials, mechanistic studies, functional magnetic resonance imaging, magnetic resonance spectroscopy, meridians, brain

## Abstract

Some cancer patients use therapeutic massage to reduce symptoms, improve coping, and enhance quality of life. Although a meta-analysis concludes that massage can confer short-term benefits in terms of psychological wellbeing and reduction of some symptoms, additional validated randomized controlled studies are necessary to determine specific indications for various types of therapeutic massage. In addition, mechanistic studies need to be conducted to discriminate the relative contributions of the therapist and of the reciprocal relationship between body and mind in the subject. Nuclear magnetic resonance techniques can be used to capture dynamic *in vivo* responses to biomechanical signals induced by massage of myofascial tissue. The relationship of myofascial communication systems (called “meridians”) to activity in the subcortical central nervous system can be evaluated. Understanding this relationship has important implications for symptom control in cancer patients, because it opens up new research avenues that link self-reported pain with the subjective quality of suffering. The reciprocal body–mind relationship is an important target for manipulation therapies that can reduce suffering.

## 1. INTRODUCTION

Therapeutic massage is increasingly used in medical treatment programs to reduce symptoms, improve coping, and enhance quality of life 1,2. Cancer patients use therapeutic massage to improve symptom control and their personal sense of wellbeing.

The largest published report on therapeutic massage is a prospective, nonrandomized, observational study of patients treated at the Memorial Sloan–Kettering Cancer Center in New York City 3. That study evaluated changes in symptom scores for pain, fatigue, stress and anxiety, nausea, and depression. Participants included 1290 cancer patients and 12 licensed massage therapists. Three variations of massage (selected mainly by the patients) were used: Swedish, light touch, and foot massage. The main outcome measures were data from symptom cards collected by independent observers that were recorded before and after the first session of massage. Symptom scores declined in severity by approximately 50%. Swedish and light touch massage were found to be superior to foot massage. However, the effects of massage were short-term.

This intriguing observational study illustrates many of the challenges in the research into therapeutic massage. The results indicated that the size of the effect for massage in cancer patients is clinically important, and the authors have since begun a randomized controlled trial.

The strength of the pilot study was the systematic collection of data from a large number of patients. Its main weakness was that it lacked a randomized control group, and therefore uncertainty remains regarding whether the intervention (massage) was the only factor that led to the improvement in the patients’ symptom scores. The patients were mainly self-selected and probably believed that the intervention would be of benefit. Symptom improvement may be a consequence of conscious belief of benefit (the placebo effect) rather than the physical manipulation or touch. In addition to the manual therapy, other ambient factors such as verbal communication, background music 4,5, and the scent of massage oils or aroma-therapy products 6,7 may have influenced outcome. The largest effect of massage therapy may be on the reduction of trait anxiety and depression, with a course of treatment providing benefits similar in magnitude to those of psychotherapy 8,9.

Currently there is a dearth of randomized controlled trials of massage therapy in cancer patients. The ones that have been reported show conflicting results that may be a consequence of variation in technique and use of non-validated symptom scores 10–13.

A recent prospective randomized trial completed by the department of radiation oncology, CHUM Hôpital Notre-Dame, and the Canadian Touch Research Centre in Montreal 14 evaluated the effects of massage therapy on anxiety levels in patients undergoing radiation therapy. In a 6-month period, 100 patients undergoing radiation therapy were randomly assigned to either massage sessions or control sessions. The massage group received a 15-minute massage session before radiotherapy over 10 consecutive days. The control group did not receive massage. The State–Trait Anxiety Inventory and a Visual Analog Scale were used to evaluate both groups.

Following massage, anxiety scores in the patients were significantly reduced (by 43%) as compared with pre-massage scores. In both groups, patients experienced an average 20% reduction in anxiety between the first and the last radiotherapy session, but that result did not reach statistical significance. The massage therapy was associated with an immediate significant decrease in anxiety scores before radiotherapy (procedural anxiety), but it appeared to have no major impact on situational anxiety. However, the period of intervention and assessment was quite short, and so no conclusions can be drawn regarding long-term outcomes.

The most recent publication of a randomized controlled trial of massage for cancer patients is a multicentre study from four U.K. cancer centres and a hospice 15. A total of 288 cancer patients, referred to complementary therapy services for clinical anxiety or depression, or both, were allocated randomly to a course of aromatherapy massage or to usual supportive care alone. Reduction in anxiety and depression was significant at 2 weeks after the intervention, but not at 6 weeks. The authors concluded that aromatherapy massage is an effective therapeutic option for the short-term management of mild-to-moderate anxiety and depression in patients with cancer. They suggested that the benefits of aromatherapy massage need to be compared with those of psychological interventions for this patient group.

To be able to design appropriate randomized controlled clinical trials, a better mechanistic understanding of therapeutic massage is required. In particular, the physiologic pathways involved need to be understood, including the connection between myofascial manipulation, blood flow, and central nervous system adaptations. Prolonged intervention with massage therapy may possibly induce more permanent neuro-physiologic adaptations because of neural plasticity.

## 2. DISCUSSION

### 2.1 Clinical Relevance of Therapeutic Massage

Massage therapy involves the administration of combinations of specific physical manipulations applied in a systematic way, with varying intensity, direction, rate, and rhythm, to the soft tissues of the body. The therapist may work within a particular theoretical model or framework, but the application of the manipulations is usually varied to fit the subject’s health status, desired outcomes, and preferences, and the therapist’s eclectic approach.

Various types of massage have evolved from various cultural traditions (Table I). The Eastern techniques are usually based on theoretical systems that involve energy movement through channels called “meridians” or energy centres called “chakras.” The classification and uniform practice of massage therapy techniques are not completely established. Validated definitions are a requirement for research protocols.

### 2.2 Massage Techniques

#### 2.2.1 Western Tradition

**Swedish massage** consists of continuous systematic strokes and deep kneading and stretching to loosen tight muscles and to reduce stress. The manual techniques specifically include *effleurage* (smooth gliding movements intended to evoke the relaxation response), *petrissage* (lifting, squeezing, wringing, or kneading of soft tissues to stimulate deep muscle and to increase circulation), *friction* (penetrating pressure with fingertips to reduce muscle spasm), and *tapotement* (rapid striking to stimulate tissues). Myofascial release techniques are employed to stretch and relax muscles that are tense or in spasm. Chronically tense muscles restrict blood flow and may be associated with fatigue. By applying specific pressure to connective tissues or fascia, normal alignment and function can be restored and chronic pain eliminated. The technique stretches and releases the fascia to release constriction and spasm, which causes pain.

**Soft-tissue release** is a technique that uses specific compression and precise extension, administered in a systematic manner, to release muscle spasm and scar tissue.

**Trigger-point therapy** (**myotherapy**) consists of stretching the myofascial tissue through sustained specific contact with pressure points, which helps to release tension and pain. Myotherapy is the diffusion of trigger points in muscles and the retraining of muscles to relieve pain. Trigger points are usually found in tight bands of muscle, which may radiate pain to other areas of the body. For instance, relieving a tense trigger point in the back could help to ease pain in the shoulder or to reduce headaches.

**Neuromuscular therapy** uses static pressure on specific myofascial points to relieve pain. This technique manipulates the soft tissue of the body (muscles, tendons, and connective tissue) and is thought to balance the central nervous system.

**Lymphatic drainage** is a very slow, light-touch, rhythmic massage that helps the body move lymph throughout the lymphatic vessels. It reduces edema and is described as removing toxins and boosting immunity.

**Craniosacral therapy** is a treatment approach that focuses on a gentle, hands-on technique used to evaluate and enhance the function of the cranial–sacral system. This hypothetical physiologic body system comprises the membranes and cerebrospinal fluid that surround and protect the brain and spinal cord. Craniosacral treatment is said to enhance the body’s natural healing processes, improving the operation of the central nervous system, dissipating the negative effects of stress, enhancing health, and strengthening resistance to disease.

**Movement re-education** uses slow, rhythmic movements and sustained stretches to help restore and increase the normal range of motion in a joint and surrounding structures, while assisting with muscle relaxation.

#### 2.2.2 Eastern Tradition

**Shiatsu,** meaning “finger pressure,” is a Japanese massage, a form of physical manipulation of acupuncture points and meridians. The latter are thought to channel vital energy. Working on the same principle as acupuncture, practitioners apply pressure to key points known as *tsubos* (Chinese acupuncture points) on the surface of the body to stimulate the flow of energy, called *ki* (*qi* or *chi* in Chinese).

The *ki* flows in meridians beneath the skin. The practitioner works with fingers, thumbs, elbows, knees, and feet along the meridians to remove *ki* blockages or overactivity (called *jitsu*), to restore areas of *ki* depletion (called *kyo*), and to stretch and mobilize limbs to facilitate the flow of *ki*. ***Tui na*** is a similar system derived from Traditional Chinese Medicine.

**Acupressure** is an ancient Asian healing art that uses the fingers on the surface of the skin to press key points that modulate energy flow through meridians and chakras. Manipulation of energy flow is speculated to stimulate the body’s immune system and enhance self-healing.

**Reflexology** consists of firm pressure to specific points on the feet, hands, or ears. Reflexology is based on the principle that these regions contain links that correspond to every other part of the body.

***Jin-shin do*** is a form of acupressure that was developed in Japan by Jiro Muraim, who mapped out a healing system based on his own body’s acupressure points and their responses to energy flow. A combination of acupressure points called “safety energy locks” is held with the fingers for a minute or more.

**Thai massage** (*nuad borarn*), is an ancient bodywork system designed to unblock trapped energy and to improve vitality by applying pressure along the meridian channels.

**Polarity therapy** is a complete system developed by Randolf Stone, a chiropractor and osteopath who believed that illness or pain in the body was cured more readily in concert with awareness and relaxation. The treatments combine therapeutic bodywork, healing intent, dietary adjustments, counselling aimed at awareness, and yoga-style exercises. The term “polarity” describes the basic nature of the hypothesized “electromagnetic force field” of the body.

### 2.3 Safety of Massage Therapy

Massage administered by a registered (or licensed) massage therapist is very safe; complications are rare 16. Healthy patients may occasionally experience bruising, swelling of massaged muscles, a temporary increase in muscular pain, or an allergic reaction to skin lubricants. Case reports have documented serious adverse events that include fractures and dislocations, internal hemorrhage and hepatic hematoma 17, dislodging of deep venous thromboses and resultant embolism of the renal artery 18, and displacement of a ureteral stent 19. Adverse effects were associated mainly with massage delivered by laypeople and with techniques other than Swedish massage.

Practitioners need to be aware of the following special situations with cancer patients:

- Coagulation disorders, complicated by bruising and internal hemorrhage
- Low platelet count
- Medications: coumadin, acetylsalicylic acid, heparin
- Metastases to bone, complicated by fracture
- Open wounds or radiation dermatitis, complicated by pain and infection

In these situations, avoiding massage or lightening the touch over regions of risk may prevent complications. No evidence suggests that massage therapy can spread cancer, although avoiding direct pressure over a tumour is a sensible precaution.

### 2.4 Qualifications of the Massage Therapist

Requirements and laws for training and licensing vary from one U.S. state to another and from one Canadian province to another. Education, experience, certification, and licensing are all important credentials. Variation in philosophy and education is typical, and some massage therapists hold the mistaken belief that cancer is a contraindication to massage.

The Commission on Massage Therapy Accreditation in the United States considers 500 hours of training to be a minimum basic requirement. If a therapist is licensed in the United States, the initials lmt (licensed massage therapist) or lmp (licensed massage practitioner) are used after the therapist’s name. In non-licensing states, a therapist should have a cmt (certified massage therapist) as the minimum qualification. The letters nctmb indicate that the therapist has voluntarily taken and passed an examination given by the National Certification Board of Therapeutic Massage and Bodywork.

In Canada, the “gold standard” for massage therapy education, as set out by the Canadian Massage Therapists Alliance, demands a minimum of 2200 hours. However, considerable diversity exists in the number of hours of education and in the curricula and the types of educational institutions across the country. Some educational institutions have articulation agreements with universities for degree completion in science at the baccalaureate level.

Increasingly, massage therapy education in Canada is embracing an evidence-informed, outcomes-based model for curricula. Massage therapy is currently a regulated health profession in Ontario, British Columbia, and Newfoundland and Labrador. In the regulated provinces, students must successfully complete written and practical entry-to-practice examinations based on standards of practice set by the regulatory body. Successful applicants are eligible to use the designation mt (massage therapist) or rmt (registered massage therapist) and to qualify for third-party insurance coverage for services. In unregulated provinces and territories, well-organized professional associations impose educational standards similar to those in the regulated provinces. Membership in provincial associations may also include title designation and access to third-party insurance coverage for services.

For massage therapists working with cancer patients, specialized education and experience is essential. Programs for advanced training in massage care of patients with cancer are integrated into undergraduate curricula in the regulated provinces in Canada, and they are also available in continuing education programs in Canada and the United States—for example, at Memorial Sloan–Kettering Cancer Center 20. Important elements include safety, communication with oncologists, and recordkeeping. Massage therapists are also urged to participate in clinical trials, and courses on research methodology are encouraged.

### 2.5 Clinical Evidence for the Effectiveness of Therapeutic Massage

The main indications for massage in general practice are back symptoms (20%), relaxation (19%), neck symptoms (17%), mood disorders (7%), and leg symptoms (4%). Therapeutic massage can be effective in treatment programs for pain. The mechanisms for reducing pain may consist of local effects on muscle and effects on the subconscious parts of the brain that control the experience of pain and emotions.

The most common current use of therapeutic massage is in back pain and sports-related injuries. In North America, back pain is reported to occur at least once in 85% of adults under the age of 50. Nearly all of these patients will experience at least one recurrence. Back pain is the second most common illness-related reason given for a missed workday and the most common cause of disability.

Back pain is non-specific in 70%–90% of cases and is associated with overuse or underuse of the back 21. It manifests as tightening or spasm of the paraspinal muscles. Inflammation and swelling often occur in the joints and ligaments. Injured muscles often meet the diagnostic criteria for the so-called myofascial pain syndrome. Myofascial pain is characterized by muscles in a shortened or contracted state, with increased tone and stiffness. They often contain trigger points (tender, firm, 3-mm to 6-mm nodules that are identified on palpation of the muscles).

The Cochrane Collaboration has reviewed therapeutic massage for non-specific low back pain 22. The authors concluded that massage therapy may be beneficial for patients with subacute and chronic non-specific low back pain, especially when combined with exercise and education.

The Cochrane Collaboration has also reviewed the role of therapeutic massage and aromatherapy for cancer-related symptoms 6. They concluded that massage or aromatherapy plus massage confer short-term benefits on psychological wellbeing, with the effect on anxiety supported by limited evidence. Effects on physical symptoms may also occur.

Available evidence is sufficient to indicate that therapeutic massage is a useful discipline for the relief of a variety of symptoms that affect both the body and the mind. Clinical trials of better design are required to determine precise indications for massage and to ascertain whether specific techniques are more beneficial than others for particular symptoms. Mechanistic studies are required to understand the psychophysiologic effects of massage and the influence of those effects on clinical practice.

### 2.6 The Neuro-myofascial Biology of Touch and Massage

#### 2.6.1 Potential Mechanisms

Therapeutic massage improves local musculoskeletal symptoms and function and can also positively affect mood state and pain threshold. The mechanisms by which massage exerts these multiple therapeutic effects are not yet known.

Manipulation of affected muscles and fascia (as in Swedish massage) induces local biochemical changes that modulate local blood flow and oxygenation in muscle. These local effects may influence neural activity at the spinal cord segmental level and could modulate the activities of subcortical nuclei that influence mood and pain perception. In addition, massage of acupuncture points away from the painful muscles, fascia, and facet joints (as in Japanese shiatsu massage) can also modulate the activities of the limbic system and subthalamic nuclei through poorly understood somatic pathways called meridians. Beneficial late effects are possible through neural plasticity and remodelling.

A meta-analysis of massage therapy research has discussed the limitations of using a medical model and suggests the use of a psychotherapy perspective 8. The authors concluded that multiple applications of massage therapy reduced delayed assessment of pain and that reductions of trait anxiety and depression are massage therapy’s largest effects, with a course of treatment providing benefits similar in magnitude to those of psychotherapy.

It is unclear whether the therapeutic benefits of massage occur primarily as a result of manipulation of muscle and ligaments, or through the brain as a result of interaction with subcortical components of the nervous system. Those components modulate autonomic functions that influence mood and the perception of pain via the limbic system and brainstem nuclei.

The multiplicity of symptoms relieved suggests that subconscious mechanisms are involved in the therapeutic effects of massage 23–25. The subconscious or subcortical effects are to be distinguished from the placebo response, which stems from conscious awareness of the procedure. The relative contributions of the body–brain reciprocal relationship have not yet been delineated.

Like acupuncture, some types of massage may influence pain when applied to acupuncture points that are distant from the perceived site of the pain. Unlike therapy applied to pain at the level of the corresponding segment of the spine or dermatome, stimulation of acupuncture points influences central nervous system activity through pathways called meridians, which seem to follow musculoskeletal fascia planes 26,27. Functional magnetic resonance scanning (fmri), positron emission tomography, and single-photon emission tomography have all demonstrated the effects of acupuncture on subcortical nuclei and the limbic system 28–33. However, the influence of massage on those locations has not yet been evaluated in the published literature.

We hypothesize that massage alleviates pain through at least two pathways. The first pathway is direct manipulation of soft tissue and its innervations at the level of the involved dermatome. Manipulation of the muscle and fascia may induce local biochemical changes (lactic acid, adenosine triphosphate and phosphocreatinine) and can modulate blood flow and oxygenation of muscle 34–36. Local changes may influence neural plasticity at the associated segmental level of the spinal cord and the release of neuropeptides (such as calcitonin gene–related peptide) that increase perfusion 37,38. Myofascial stretching may transduce into electrophysiologic activity that can reduce pain and other symptoms through both a myofascial communication system and afferent neural pathways that modulate the subcortical nuclei and limbic system in the brain 39.

When a peripheral source of pain persists, intrinsic mechanisms that reinforce nociception influence the pain. Chronic pain may be seen as part of a central disturbance accompanied by disinhibition or sensitization of central pain modulation. For example, patients with chronic whiplash syndrome may have a generalized central hyperexcitability from a loss of tonic inhibitory input, contributing to dorsal horn hyperexcitability 40.

Transduction is the process whereby noxious afferent stimuli are converted from chemical to electrical neural messages in the spinal cord that communicate cephalad to the brainstem, thalamus, and cerebral cortex. Noxious mechanical, thermal, and chemical stimuli activate peripheral nociceptors that transmit the pain message through lightly myelinated A-delta fibres and unmyelinated C-fibres. Nociceptors are present in the outer annular fibrosis, facet capsule, posterior longitudinal ligament, associated muscles, and other structures of the spinal motion segment. Nociceptive modulation first occurs in the dorsal horn, where nociceptive afferents converge to synapse on a single dorsal root neuron. Hyperalgesia and allodynia initially develop at the injury site. However, when central sensitization occurs, the area of pain expands beyond the initial region of tissue pathology. Attachment to emotion may increase the perception of pain and could conceivably translate into exacerbation of somatic symptoms 23–25,41,42. Pain is motivational and is not only a conscious somatosensory perception but also a motivational feeling attached to the limbic system 43.

Swedish massage may have a direct effect primarily on muscle physiology and metabolism that, in turn, may communicate with the central nervous system through the dorsal horn afferents at the particular dermatome level. In turn, spinothalamic fibres may later activate subcortical nuclei. On the other hand, by manipulating acupuncture points that lie on meridians, shiatsu massage may initially activate sub-thalamic nuclei that can reduce pain and combat other symptoms through both subcortical gating and modulation of the limbic system. Needling of acupuncture points *away* from a painful muscle may have a similar effect on reducing muscle pain through undefined mechanisms 44. Studying time-dependent changes in the pain behaviour of low back tissues following massage therapy would provide valuable information to compare with time changes associated with mechanisms within the subcortical brain and the spinal segmental level 45.

#### 2.6.2 Noninvasive Techniques to Evaluate the Neuro-myofascial Biology of Touch and Massage

Magnetic resonance spectroscopy (mrs) and fmri are powerful, noninvasive, non-radioactive techniques that may be used to evaluate the biology of manual therapies 46. These techniques are based on the mechanics and theory of nuclear magnetic resonance (nmr). Signals can be detected only from atomic nuclear species having the quantum mechanical property of spin. The ^1^H hydrogen atom is the most abundant of these. It provides the signal for routine mri scanning, which produces images using the contrast of water and fat. The mrs technique measures levels of particular chemical species within an acquired tissue volume. It is especially useful for evaluating the physiology of myofascial tissue.

Currently the nuclei of greatest interest are ^1^H, ^13^C, and ^31^P. Techniques that can be used to evaluate muscle physiology include

- ^1^H mrs of myoglobin to assess the intracellular partial pressure of oxygen (*p*O_2_),
- ^31^P mrs to assess metabolic capacity, and
- the combination of ^31^P chemical shift imaging to assess local metabolic demand (oxygen uptake:*V*O_2_).

Blood oxygenation level–dependent (BOLD) fmri can be used to image the neural correlates of touch and pain within the subcortical nuclei of the brain. This technique allows for indirect estimation of neural activity by detecting local hemodynamic changes, which are closely related to the integrated synaptic activity of nerve cells under physiologic circumstances 46–48.

The pathways and neural centres involved in processing information from low-threshold mechanoreceptors of the skin, carried by fast-conducting myelinated afferent fibres, have been extensively investigated in nonhuman primates. Various cortical regions, including the anterior parietal cortex (primary somatosensory cortex), the lateral and posterior parietal cortices, and motor-related areas responding to mechanical stimuli have been identified 49. Humans appear to have an expanded somatosensory cortical network. Brain regions showing increased activity during vibrotactile input and tactile recognition extend beyond the parietal lobe to include portions of the frontal, cingulate, temporal, and insular cortices 50. Available evidence suggests that the central correlates of tactile stimuli vary according to their hedonic qualities. Pleasant touch induces greater activation in the medial orbitofrontal cortex than does more intense, but affectively neutral tactile stimuli 51. Additional areas activated by pleasant but not by neutral stimuli include a rostral portion of the midcingulate cortex and an area in or near the amygdala. These findings begin to identify parts of the limbic system that may underlie emotional, hormonal, and affiliative responses to skin contact.

The forebrain pain system partly overlaps structures involved in processing non-noxious input, but painful stimuli induce higher fmri signal increases than non-noxious stimuli do. A direct comparison between the cortical correlates of touch and pain using event-related fmri showed that, besides common activations in the contralateral postcentral gyrus and parietal operculum, pain is associated with stronger involvement of the contralateral midanterior insula, anterior portion of the midcingulate cortex, and dorsolateral prefrontal cortex 52,53.

The autonomic responses to acute pain exposure usually habituate rapidly; the subjective ratings of pain remain high for more extended periods of time. Thus, systems involved in the autonomic response to painful stimulation—for example the hypothalamus and the brainstem—would be expected to attenuate the response to pain during prolonged stimulation. Areas in the brainstem are involved in the initial response to noxious stimulation, which is also characterized by an increased sympathetic response.^54^ The perigenual anterior cingulate gyrus is a crucial location for integrating cognitive, emotional, and subconscious activities in the affective dimension of pain 55,56. Pain-related modulation of fmri signals in other regions involved in reward and emotion circuitry, such as the nucleus accumbens–ventral striatum and the orbitofrontal cortex, has also been demonstrated 51. Evidence for amplified processing of mechanical stimuli in parietal, insular, and cingulate cortices has been obtained in patients with fibromyalgia, who show characteristically lowered pain thresholds. These studies have begun to shed light on the neural systems involved in central sensitization of nociceptive circuits in pathophysiologic conditions 57,58.

The relative role of cognitive awareness versus subcortical modulation may be deciphered by using distraction and attention methodologies during an fmri examination 58–63. Attentional effects may be exerted at various levels of the somatosensory system and involve activation of brainstem modulatory centres 62,64.

In a study that employed covariation analysis, a functional interaction was found between the orbitofrontal cortex and perigenual anterior cingulate gyrus, the periaqueductal gray matter and posterior thalamus during pain stimulation and distraction, but not during pain stimulation *per se* 61. Placebo-induced anticipation of pain relief treatment decreases brain activity in pain-related brain regions 65.

When evaluating the physical effect of massage, psychophysiologic techniques to discriminate between conscious attention and subconscious neurologic interaction are important. The brain networks underlying somatosensory perception are complex and highly distributed. A deeper understanding of perceptual-related and subconscious brain mechanisms therefore requires new approaches suited to investigate the spatial and temporal dynamics of activation in various brain regions and the functional interaction of those regions.

The development and application of refined tools for evaluating functional connectivity between neural populations will provide new insights into bottom-up and top-down mechanisms in somatosensory perception 53. Current evidence from fmri suggests that positive and negative tactile stimuli are both represented in the orbitofrontal cortex. The brain region in or near the amygdala is activated by pleasant touch. Most studies of the amygdala have tended to concentrate on its role in negative emotions, such as fear, but other imaging studies have found amygdala activation in response to affectively positive stimuli 51.

Therapeutic massage may transduce mechanical signals through skin sensation, proprioception, and non-noxious muscle perception 60. How this process translates into local electrophysiologic and chemical changes within muscle and fascia is not clear. Similarly, how therapeutic massage interacts with the central nervous system is not known, although some leads are emerging from research on touch. Preliminary physiologic investigations of muscle and the brain using nmr techniques suggest that therapeutic massage may have distributed effects that can reduce various unpleasant symptoms.

## 3. CONCLUSION: CHALLENGES FOR THERAPEUTIC MASSAGE RESEARCH

The mechanistic links between manipulation of body tissues and corresponding relief from a broad range of symptoms are not fully understood. The effects are distributed, and reciprocal interplay between the body and mind is evident. We have literally just “touched” the surface of meridian research, but the meridian system appears to be an important communication link between myofascial tissue and the nervous system. This traditional communication system appears to link biochemical, electrical, and physiologic changes in the myofascial tissue with subcortical neurologic activity and changes in cognitive experience. The implications for symptom control in cancer patients are important, opening up new research avenues that link self-reported pain with the subjective quality of suffering. The reciprocal body–mind relationship and its manipulation is an important target for therapies that can reduce suffering.

The U.S. National Center for Complementary and Alternative Medicine held a conference titled The Biology of Manual Therapies during June 9–10, 2005, at the National Institutes of Health (nih) in Bethesda, Maryland 66. The goal was to define three to five of the most critical research questions involved in gaining an understanding of the biology of manual therapies. Table II outlines the research recommendations. Table III lists current clinical trials involving massage and cancer (found by searching the nih clinical trials database at clinicaltrials.gov). At June 2006, seven studies investigating the effects of massage therapy in cancer patients were registered and active.

More work is required on the methodology for conducting clinical trials of therapeutic massage. Validation of the massage technique is essential. In clinical practice, both the site of massage and the technique may vary according to the practitioner’s personal judgment. In an intent-to-treat study, such variation may be valid, but excellent records should be kept to determine that the therapy was within acceptable degrees of freedom. When comparing various massage-therapy techniques, rigorous validation of the practitioners’ interventions is necessary. The design of sham massage is challenging. The control may involve touching non-therapy sites only, using untrained volunteers, providing education only, or employing a waiting list control. In addition, because thoughts of intent to heal are considered important, sham therapists may be asked to use personal distraction techniques. Defined subject populations (with appropriate inclusion and exclusion criteria) and validated outcome scales are essential.

In addition to the manual therapy itself, ambient factors such as environment, music, and aroma can influence outcome. The objectivity of research is complicated by the relationship and transference between therapist and client. The possibility exists that benefits may come about more from factors such as the recipient’s attitude toward massage therapy, the therapist’s personal characteristics and expectations, and the interpersonal contact and communication that take place during treatment than from the specific form of massage therapy used or the site to which it is applied.

Only a combination of mechanistic research and well-designed clinical trials will clarify the reciprocal relationship between body and mind and will determine the utility of manual therapies for symptom control in cancer patients.

## Figures and Tables

**TABLE I:** Taxonomy of therapeutic massage

Western tradition	Swedish
	Myofascial
	Soft-tissue release
	Trigger-point (myotherapy)
	Neuromuscular
	Reflexive
	Circulatory, lymphatic
	Craniosacral
	Movement re-education
Eastern tradition	Shiatsu
	*Tui na*
	Acupressure
	Reflexology
	*Jin shin-do*
	Thai massage
	Polarity therapy

**TABLE II:** Recommendations from the Conference on the Biology of Manual Therapies, National Institutes of Health, National Center for Complementary and Alternative Medicine; Bethesda, Maryland; June 9–10, 2005

General questions relating to mechanisms of action for manual therapy. Determine the effects of manual therapy in normal experimental animals and in animal models of tissue injury, including behavioural responses to painful stimuli, fibroblast response, gene expression. Does applying very superficial manual therapies, such as light massage, that mainly activate skin afferents produce different effects on the nervous system, immune system, and endocrine system compared with manual therapies that also involve activation of muscle afferents? Does paraspinal tissue have any unique physiology compared to appendicular tissues? Is this related to the reported clinical efficacy of manual therapies? Do manual therapies produce long-lasting changes in the biomechanics of the spine, torso, or limbs? Are these changes associated with altered activity in the nervous system? Immune system? Endocrine system? Identify valid, reliable biomechanical measures (for example, posture, kinematics, kinetics, functional imaging) that can be used to distinguish between healthy and non-healthy tissues. subcategorize patients/clients with musculoskeletal disorders. Develop imaging techniques that can be used to capture dynamic *in vivo* responses to biomechanical signals in healthy and non-healthy tissues. Questions relating to peripheral mechanisms of action for manual therapy. Determine and compare the discharge characteristics (that is, the pattern or frequency of action potentials) of primary sensory neurons in response to various types of manual therapies (for example, high-velocity loading compared with slower loading rates). Is there any correlation with reported efficacy? How do various manual therapies affect peripheral nerve biomechanics? What path of mechanical load transmission do various manual therapies take through the body? Questions relating to central mechanisms of action for manual therapy. Determine how different types of manual therapies affect the signalling properties of neurons in the central nervous system or autonomic nervous system. That is, do they produce long-lasting changes? Do different types of manual therapies evoke different patterns of neural activity in the central nervous system or autonomic nervous system? Determine effects of peripheral mechanical stimuli (for example, manual therapies) on spinal cord gating mechanisms and synaptic plasticity. Develop and use human models of experimental pain to determine the role of the nervous system, if any, in explaining how manual therapies work. Specific areas of investigation could include the effects of temporal summation, the effect of manual therapies on windup, quantitative sensory testing. Non-neural outcomes might include heart rate and heart rate variability, laser Doppler blood flow and blood pressure changes, respiratory frequency, CO_2_ levels, catecholamine levels, circulating cells (numbers/subsets/response), cytokines, vaccine response (immunoglobulin response), contact hypersensitivity, C-reactive protein, lymphatic flow.

**TABLE III:** Current North American clinical trials involving therapeutic massage and cancer patients

Phase ii randomized pilot study of massage therapy in patients with cancer pain Memorial Sloan–Kettering Cancer Center, New York, New York Principal investigator: Barrie R. Cassileth phd (telephone: 646-227-2149) To determine whether the effects of massage therapy in patients with cancer pain are sufficiently promising to warrant a definitive trial Interventions: Arm i (massage therapy): Patients receive a light touch (“Reiki”) massage over 45 minutes Arm ii (volunteer visit control): Patients receive a 45-minute visit from a trained volunteer who will be available to sit quietly or talk with the patient to discuss issues of concern, as desired by the patient. Volunteers will not touch the patient except to pat their shoulder or briefly hold their hand. Arm iii (quiet time control): Patients receive 45 minutes of quiet time. Pain and mood are assessed at baseline, immediately after treatment, at 6 hours and 24 hours after treatment, and then daily for the next 5 days after treatment. Randomized study of hypnosis, massage therapy, and healing touch in patients undergoing chemotherapy for ovarian epithelial or primary peritoneal cavity cancer. University of Minnesota Cancer Center, Minneapolis. Minnesota Chair: Patricia L. Judson md (telephone: 888-226-2376) Interventions: Arm i (standard therapy): Patients undergo standard chemotherapy for ovarian epithelial or primary peritoneal cancer. Arm ii (standard therapy with complementary alternative medicine): Patients undergo chemotherapy as in arm i. Patients also undergo massage over approximately 30 minutes and healing touch therapy over approximately 30 minutes once during courses 1–6, and hypnosis once over 30–60 minutes during courses 1, 2, and 4. Primary aim: To determine whether quality of life is improved in patients with ovarian epithelial or primary peritoneal cavity cancer receiving hypnosis, massage therapy, and healing touch and standard chemotherapy as compared with patients receiving standard chemotherapy alone. Secondary aim: To determine changes in immunologic response markers, chemotherapy side effects, and complication rates in the patients. A randomized study of polarity or massage therapy to reduce fatigue in breast cancer patients during radiation therapy. University of Rochester, Rochester, New York Principal investigator: Karen Mustian phd (telephone: 585-275-0690) A randomized three-arm clinical trial of an intervention examining the efficacy of polarity therapy for the relief of fatigue associated with radiation treatments in breast cancer patients. Patients who meet the eligibility criteria and who have signed consent will be randomized to one of three trial arms: Polarity treatment Massage treatment Standard care Three treatments will be administered in the 4th, 5th, and 6th calendar weeks of radiation treatment. Weekly blood draws will assess cytokine levels. In addition, 6 saliva samples will be gathered per day for 2 days of each of the 4 study weeks to assess cortisol levels. An actigraph will be worn for the 28 study days to assess activity and sleep. Patients randomized to the standard care arm will receive a polarity or massage treatment gratis following the completion of the study. Primary outcomes: Fatigue, subjectively by the Brief Fatigue Inventory and the Multidimensional Fatigue Symptom Inventory and objectively by actigraphy; mood by the Fatigue/Inertia subscale of the Monopolar Profile of Mood States. Secondary Outcomes: Health-related quality of life (Functional Assessment of Chronic Illness Therapy–Fatigue); quality of sleep assessed subjectively with the Pittsburgh Sleep Quality Inventory and a sleep diary. Does scar massage improve postoperative pain and function in women with breast cancer? A randomized controlled study. University of British Columbia (BC Cancer Agency), Victoria, British Columbia Principal investigator: Pauline Truong md (telephone: 250-519-5512) A prospective randomized controlled trial design involving women who have undergone breast cancer surgery. Subjects will be randomized into two cohorts: scar massage (intervention group) and no scar massage (control group). Primary outcomes: Scar-related pain (scored using the McGill Pain Questionnaire–Short Form) Secondary outcomes: Upper-body range of motion, physical parameters of the scar (pliability, scar height, vascularity, and pigmentation scored using the Vancouver Scar Scale), lymphedema (evaluated by arm circumference measurements), and quality of life. 5. Massage therapy for breast cancer-related lymphedema. University of Arizona, Tucson, Arizona Principal investigator: Marlys Witte md (telephone: 520-626-6118) Randomized, single-blind, active-control, parallel-assignment efficacy study Patients will be randomly assigned to either treatment with manual lymph drainage alone or a combination of manual lymph drainage and compression bandaging. Patients will be treated in 10 one-hour sessions over 2 weeks. They will also undergo lymphangioscintigraphy to depict the function of their lymphatic system. Patients will continue self-treatment at home and will be followed for 6 months. Purpose is to examine the short-term and long-term efficacy of massage therapy alone as compared with massage therapy plus compression bandaging in the treatment of breast cancer treatment–related swelling of the arms and legs. delta: A randomized trial of decongestive lymphatic therapy (dlt) for lymphedema in women with breast cancer. Ontario Clinical Oncology Group, Hamilton, Ontario Principal investigator: Ian Dayes md (telephone: 905-527-2299, ext. 42610) Randomized patients receive either standard of care, or standard of care plus dlt (five massage sessions per week during 4 consecutive weeks). Primary evaluation of all patients is recorded 6 weeks after randomization by measuring the affected limb and comparing with the unaffected one. Extended follow-up of one year will be conducted. Primary outcomes: Percent reduction in excess arm volume at 6 weeks as calculated from circumferential arm measurements. Secondary outcomes: Measurement of arm function, quality of life rest (reducing end-of-life symptoms with touch): Efficacy of massage at the end of life. University of Colorado, Denver, Colorado Principal investigator: Jean S. Kutner md (telephone: 303-372-9088) Participants will be randomly assigned to receive 6 sessions of either moving or non-moving touch therapy, in addition to usual hospice care, for 2 weeks. Moving touch therapy consists of massage therapy in which a trained therapist continually touches a person’s body. The non-moving touch therapy will be conducted by volunteers who have no previous experience in massage. Participants in this group will have a volunteer rub specific body parts for three-minute intervals. Because current evidence suggests that thoughts of healing may influence the effectiveness of touch therapy, volunteers in the non-moving therapy group will distract their minds to avoid thinking of healing processes. In both groups, the person administering the touch therapy will note all interruptions during a session, including talk, music, and television. Interviews about medication use, pain, and quality of life will be used to assess participants; these interviews will be conducted at study start, immediately before and after each therapy session, and at weeks 1, 2, and 3. Primary outcome: Decreased pain level. Secondary outcomes: Less total analgesic medication use, improved quality of life, decreased physical symptom distress, decreased emotional symptom distress
